# Mental health differences in medical students based on curriculum and gender

**DOI:** 10.1186/s12909-023-04946-2

**Published:** 2023-12-19

**Authors:** Maxim Jestin, Shelly Sharma, Deval Jhaveri, Brittany Mitchell, Dean Micciche, Venkat Venkataraman, Kathryn Lambert

**Affiliations:** Rowan-Virtua School of Osteopathic Medicine, Stratford, NJ USA

**Keywords:** Medical students, Emotional exhaustion, Anxiety, Depression, PBL, LBL, Curriculum

## Abstract

**Background:**

The prevalence of mental health struggles among students in medical school is widely reported; however, little is known about how it is impacted by the medical school curriculum. This study aimed to evaluate differences in anxiety, depression, and emotional exhaustion in medical students based on gender, class year, and curriculum.

**Methods:**

An anonymous online survey consisting of questions from established, validated questionnaires about demographics, anxiety, depression, emotional exhaustion, and personal health behaviors was sent to 817 medical students who attended Rowan-Virtua School of Osteopathic Medicine during the Spring of 2021. When applying to this school, each of these students had the option to choose either the problem-based learning (PBL) or lecture-based learning (LBL) curriculum track.

**Results:**

The survey was completed by 222 students. Females experienced higher levels of anxiety, depression, and emotional exhaustion than males. Students in the PBL had lower levels of emotional exhaustion than their peers in the LBL. Increase in emotional exhaustion was most pronounced between 1st and 2nd year students. Emotional exhaustion was inversely correlated with sleep and exercise.

**Conclusions:**

On average, students who were either male or in the PBL curriculum experienced less mental distress in the form of anxiety, depression, and emotional exhaustion than their peers. While gender continues to be an established factor in how mental distress is experienced, the reduced levels of emotional exhaustion in PBL students is a novel finding that can potentially shed light on how to better optimize medical education. Despite the inherent selection bias and lower number of PBL students, to our knowledge, this is the first study comparing two different curricula within a single institution. This finding along with a focus on good sleep and exercise habits may provide a path for improving mental health in medical students.

## Background

The field of medicine tends to attract individuals who exhibit perseverance [1, 2]. Unfortunately, this same quality may lead to burnout, which is characterized as emotional exhaustion, depersonalization, and reduced sense of personal accomplishment [3]. The repercussions of burnout are far-reaching, affecting both the individual and the system in which they operate. A prime example is physician burnout which has an estimated prevalence of around 50%, a rate markedly higher than professionals in other fields [4, 5]. Physician burnout can have personal consequences, such as dissolution of relationships, suicidal ideation, and problematic drug and alcohol use, as well as professional consequences, which include erosion in quality of care, increased risk of medical error, and early retirement [6, 7]. This leads to a significant cost to the healthcare system, estimated at $4.6 billion each year nationally when taking into account reduced clinical hours and physician turnover [8].

The onset of burnout is insidious, often starting early in training. Among medical students, about half express feelings of burnout and 1 in 9 report suicidal ideation [9]. While reported rates of suicidal ideation decrease among residents/fellows and early career physicians, the rates of burnout remain relatively constant [10]. Burnout early in medical training can have dire consequences. Students become more susceptible to depression, leading to impaired concentration and apathy that may hinder their ability to pass courses. This increases their risk of dropping out of medical school and potentially distancing themselves from the profession entirely. Furthermore, students with burnout are more inclined to engage in unethical behaviors and less likely to hold altruistic views about their responsibility to society as a future physician [11]. The depersonalization aspect of burnout may cause students to be less empathetic toward patients, viewing them as a set of symptoms rather than a person, which can lead to problems with medical errors later in their training [1].

While medical student distress in the form of anxiety, depression, and burnout has been well documented in the literature, [9–14] there is a scarcity of studies examining the impact of curriculum on mental health. The traditional, lecture-based learning (LBL) curriculum has long been the standard in medical education; however, problem-based learning (PBL), sometimes referred to as a case-based learning curriculum, has seen increased implementation among U.S. medical schools in recent decades. In a PBL curriculum, students attend small peer group sessions where they are presented with a problem or case, on a subject matter they cover independently prior, to discuss in the presence of an instructor. This format encourages critical analysis of a variety of perspectives, fosters a deeper understanding of the content, and promotes self-directed learning habits [15–19]. Several studies suggest improved academic performance and perceptions of learning among students in PBL compared to their peers in LBL [20–22]. However, there is limited literature on the impact of the two differing curriculum styles on medical student mental health. The unique structure of the Rowan-Virtua School of Osteopathic Medicine (Rowan-Virtua SOM) provides both LBL and PBL curricula to accepted students. When applying, students can choose either the LBL or PBL track, thus students self-select into their respective curriculum. Notably, the PBL track has limited seats, meaning not all accepted students get their preference and therefore not all students who choose the PBL curriculum may be part of it; instead, they are placed in the LBL track if no more PBL seats are available. We aimed to investigate curriculum differences by disseminating a mental health survey assessing common elements of psychological distress among medical students. Another factor of interest is gender. Earlier studies have suggested gender as an important factor in burnout: nearly twice as many female medical students displayed impostor syndrome associated with burnout compared to their male counterparts [13]. In another study utilizing the Depression and Anxiety Stress Scale scoring, female gender was strongly associated with anxiety as well as depression [14]. However, this impact of gender may not be unique to medical students – an epidemiological study found that women are approximately 1.7 times as likely as men to report a lifetime history of major depressive episodes [23]. Finally, we investigated potential personal health behaviors of influence such as sleep and exercise. Lack of sleep and exercise are other factors that potentially play a significant role in increasing burnout risk and stress among medical students [24]. This study seeks to explore the impact of gender and curriculum on anxiety, depression, and emotional exhaustion, a key component of burnout, as students progress through medical school, and evaluate the role of beneficial behaviors such as adequate sleep and exercise.

## Methods

In this cross-sectional study, an anonymous web-based survey utilizing Qualtrics, an online surveying platform, was emailed to all 817 medical students at Rowan-Virtua SOM. Of note, students generally self-select into the LBL or PBL curriculum; there is some competition for the PBL curriculum due to limited seats. Due to students having the option of PBL or LBL, there is an inherent selection bias. Recruitment emails were sent between the end of March and start of June of the 2021 academic year during a time specific to each class cohort’s schedule when students would be most available. Participants provided consent on the initial page prior to starting the survey. Survey responses were tracked anonymously using Qualtrics and reminders were sent to non-respondents every two weeks, with the last reminder being sent two weeks prior to the survey closing. The survey was advertised as an anonymous, voluntary 10–15 min survey on mental health. Students were not required to complete all sections of the survey for submission. Data was collected and stored on Qualtrics. As an incentive, participants were offered a chance to win one of the four $25 Amazon gift cards (one for each class year) by participating in a separate, post-survey raffle hosted on Google Forms.

## Survey measures

The survey is a modified version of the mental health survey used by Gold et al., [25] with their permission. Results collected and analyzed were derived from the following sections: (i) Demographics, (ii) Stress and Burnout, and (iii) Personal Health and Coping Behaviors. The questions were formatted using the Likert scale rating. A modified version of the Maslach Burnout Inventory limited to emotional exhaustion questions, served as our assessment for burnout, as emotional exhaustion is a strong predictor for burnout [26].

Mental health was assessed using the Patient Health Questionnaire-4 (PHQ-4), a brief and validated screening scale for anxiety and depression [27]. Anxiety and depression were assessed individually through subscales (positive if score ≥ 3) or as a composite score: normal (0–2), mild (3-5), moderate (6-8), and severe (9-12), denoted as mood disorder in this manuscript. Personal health behaviors were assessed with questions about sleep and exercise.

### Statistical analyses

Statistical comparisons were carried out using Graphpad Prism v10.0 and/or SPSS v28. Graphical representations of the data were created using Graphpad Prism v10.0. Pearson’s chi-squared test and independent t-test were utilized to compare the differences between data sets and a value of *p* < 0.05 was considered statistically significant.

## Results

### Demographics

Of the 817 students to whom the survey was sent, 222 students completed the entire survey. The composition of respondents included 45 from the PBL curriculum (20%) and 177 from the LBL curriculum (80%), aligning closely with the PBL to LBL curriculum distribution at Rowan-Virtua SOM (22% vs. 78%, respectively). Of the students who completed the survey, 122 (55%) were female and 100 (45%) were male. Overall, Rowan-Virtua SOM is composed of 50% females and 50% males. Respondents included 77 first year students (class of 2024; 35%; 19 PBL, 58 LBL), 55 s year students (class of 2023; 25%; 7 PBL, 48 LBL), 66 third year students (class of 2022; 30%; 14 PBL, 52 LBL), and 24 fourth year students (class of 2021; 11%; 5 PBL, 19 LBL).

### Anxiety, Depression, and emotional exhaustion

**Fig. 1:**
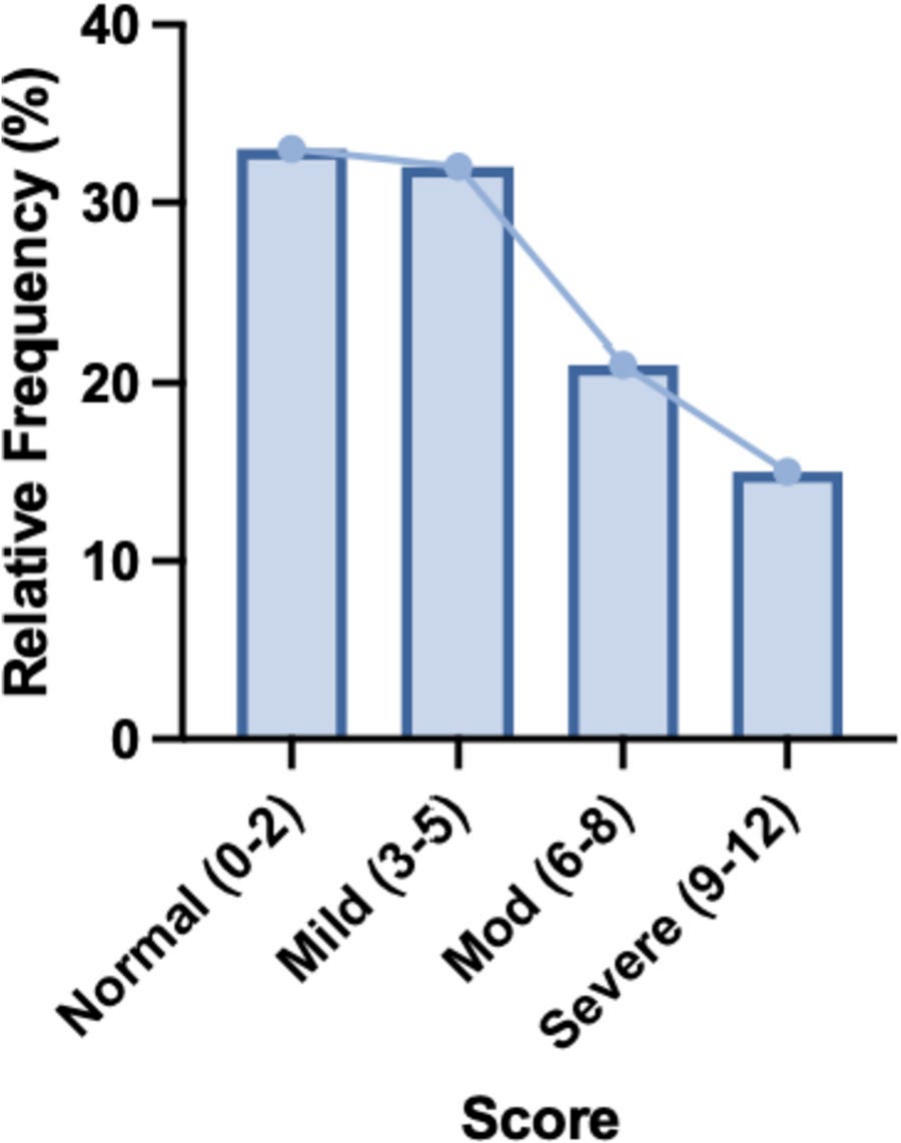
Mood disorder among all students. Frequency of PHQ-4 composite scores

**Fig. 2:**
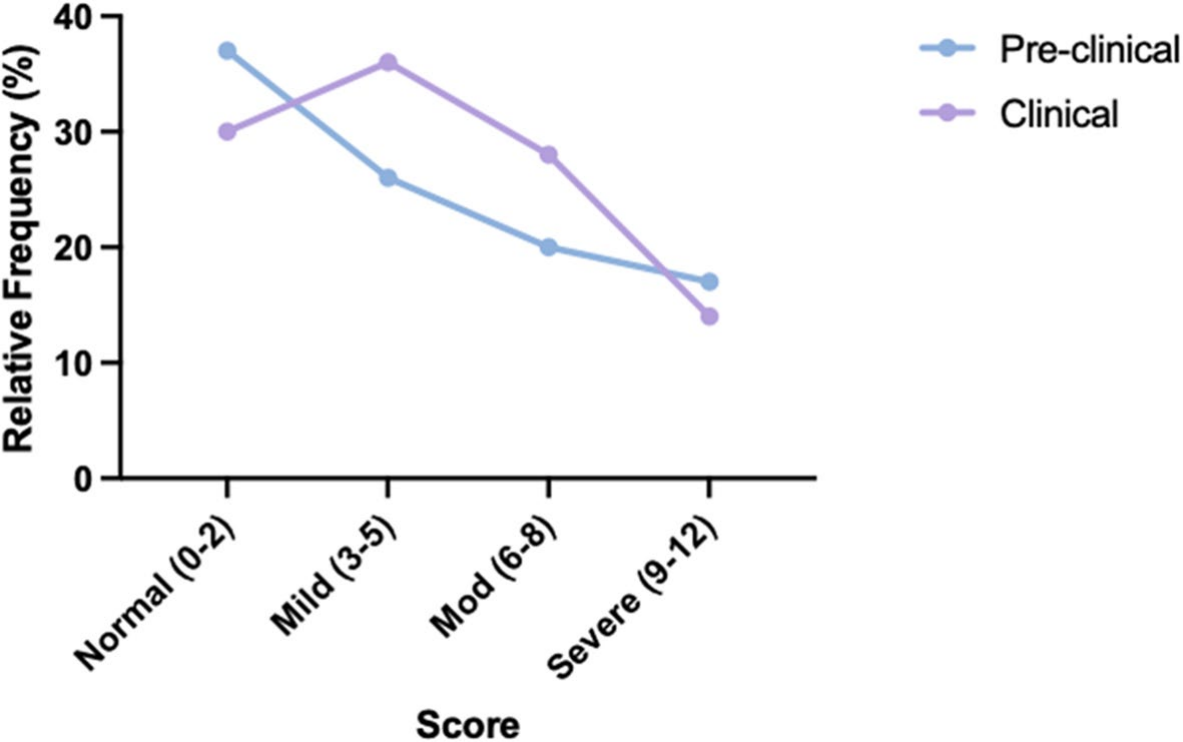
Comparison of mood disorder between pre-clinical and clinical cohorts. Frequency of PHQ-4 composite scores

**Fig. 3:**
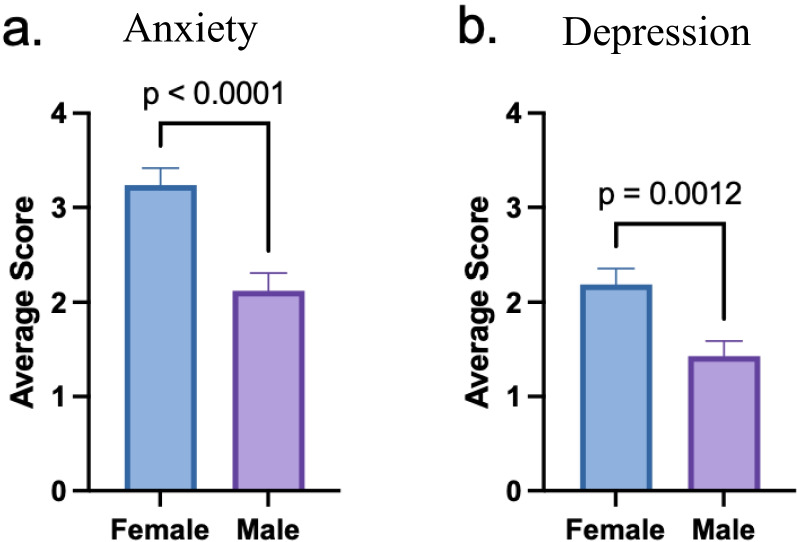
Comparison of anxiety and depression by gender. **a** Average anxiety subscale scores. **b** Average depression subscale scores

**Fig. 4:**
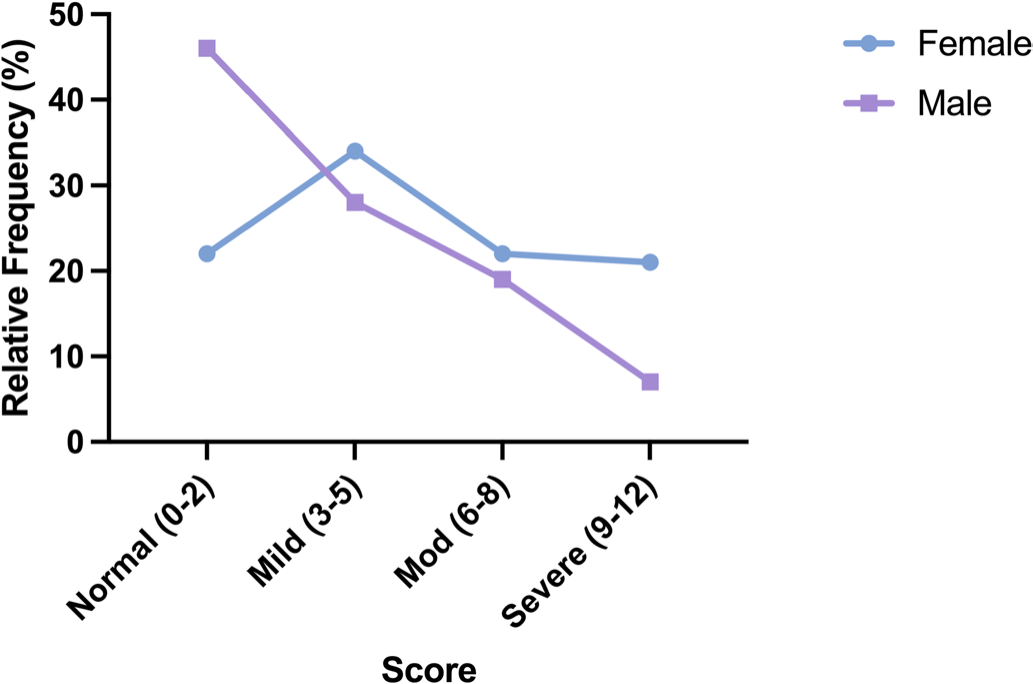
Comparison of mood disorder by gender. Frequency of PHQ-4 composite scores

Out of the 222 responses, 79 (36%) met the criteria for moderate or severe mood disorder based on a composite score on the PHQ-4 questionnaire (Fig. 1). According to the PHQ-4 subscales, 109 (45%) students screened positive for anxiety and 60 (27%) screened positive for depression. Levels of anxiety and depression did not differ significantly from one class year to another (data not shown). When grouped by preclinical (1st /2nd ) and clinical (3rd /4th ) years, the mood disorder distribution was similar between cohorts (Fig. 2). On average, females reported significantly higher levels of anxiety and depression than males (Fig. 3). This difference was reflected in the frequency distribution showing that a greater percentage of females have a higher degree of mood disorder compared to males on the composite PHQ-4 scale (Fig. 4).

**Fig. 5:**
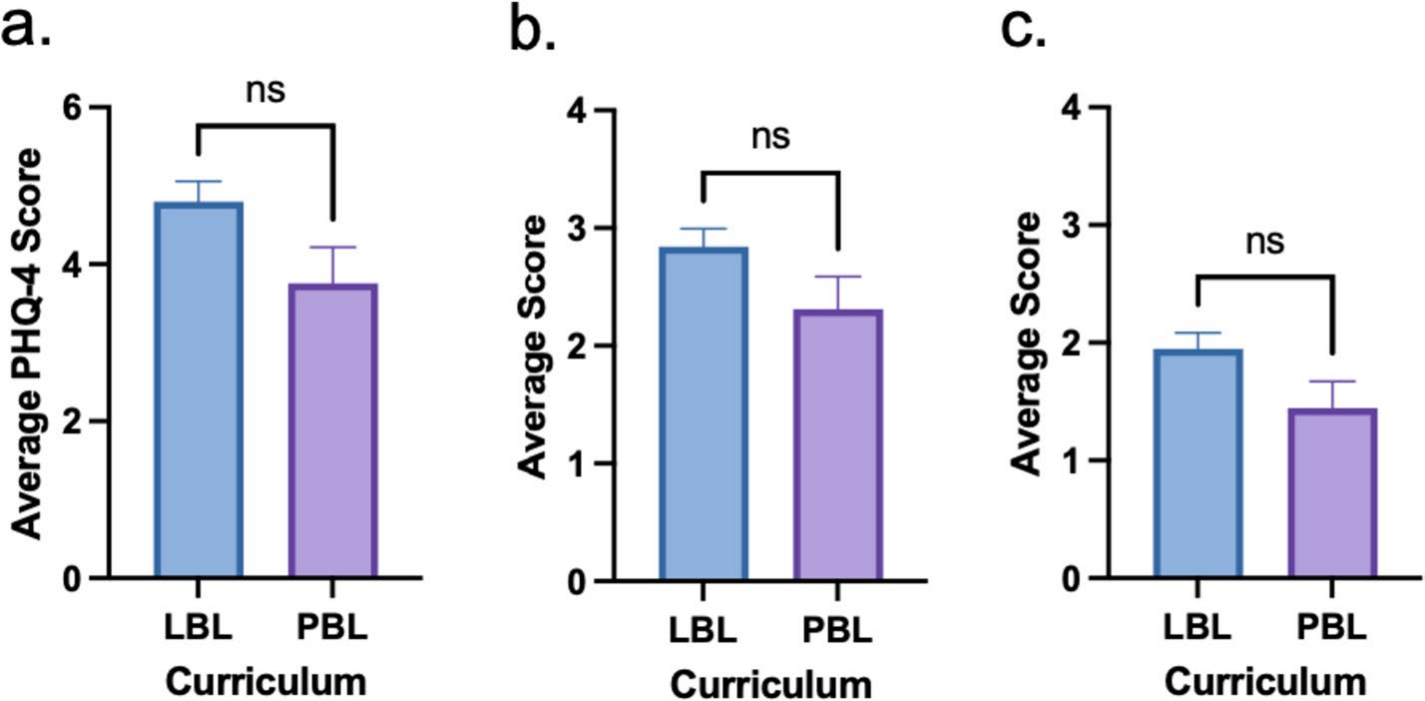
Comparison of anxiety and depression by curriculum. **a** Average PHQ-4 composite scores. **b** Average anxiety subscale scores. **c** Average depression subscale scores

**Fig. 6:**
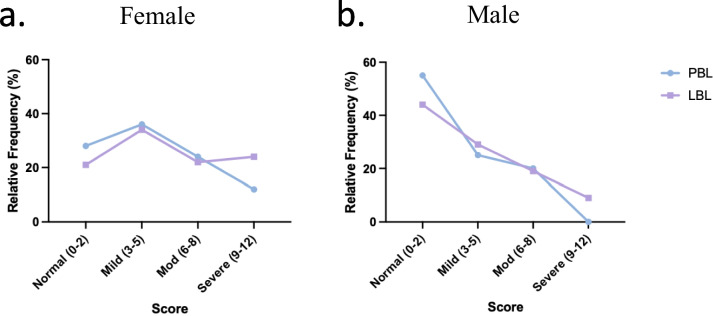
Comparison of mood disorder by curriculum and gender. **a** Frequency of PHQ-4 composite scores among females in PBL and LBL. **b** Frequency of PHQ-4 composite scores among males in PBL and LBL.

A curriculum comparison showed that PBL students tend to have lower levels of anxiety, depression, and general mood disorder than LBL students, but this was not statistically significant (Fig. 5). Factoring in gender into the curriculum comparison of mood disorder showed no significant difference (Fig. 6).

**Fig. 7:**
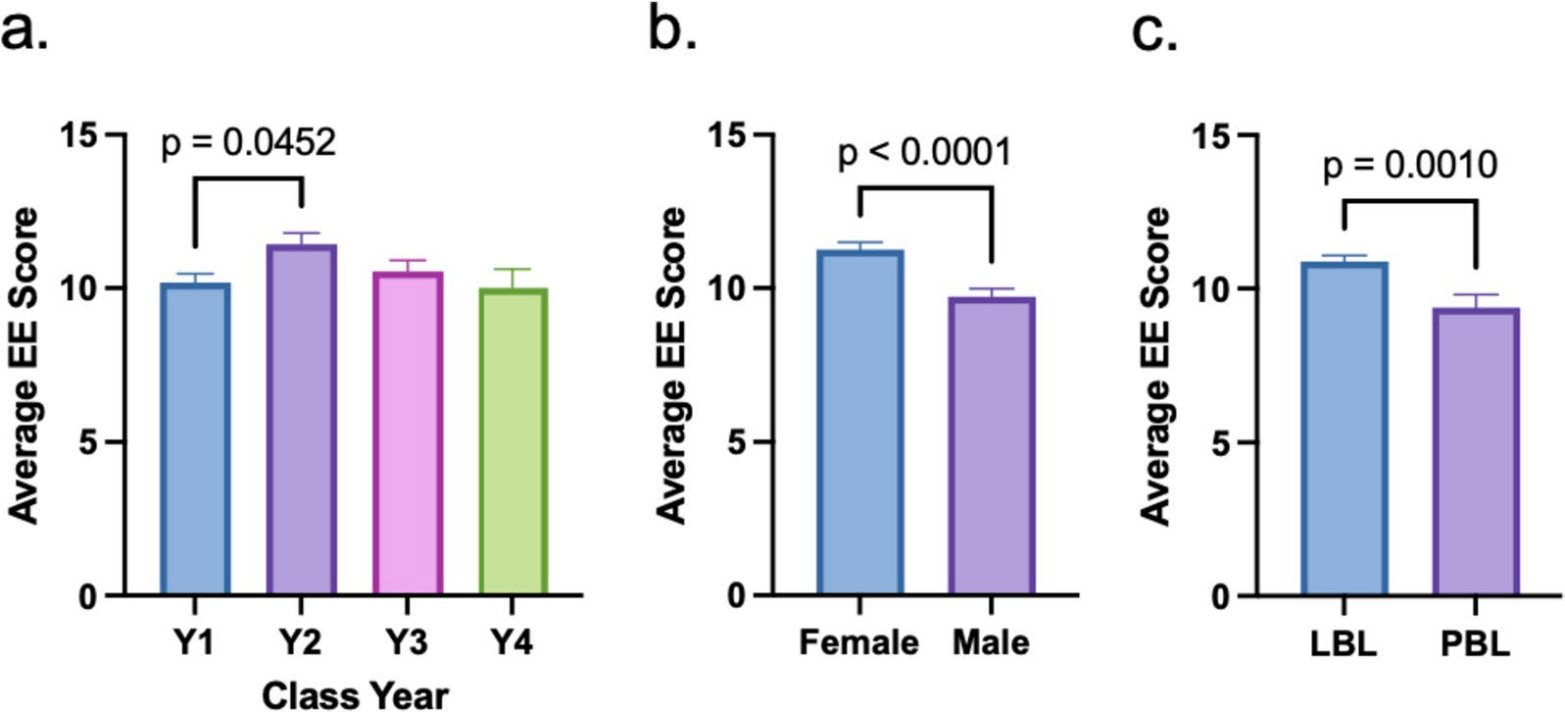
Comparison of emotional exhaustion (EE). **a** Average EE scores by class year. **b** Average EE scores by gender. **c** Average EE scores by curriculum

Degree of emotional exhaustion did not change significantly between preclinical and clinical cohorts; however, a significant increase was noted between first year and second year students (Fig. 7a). A gender comparison demonstrated higher levels of emotional exhaustion in females than males (Fig. 7b). When comparing curricula, LBL students showed higher levels of emotional exhaustion than PBL students (Fig. 7c).

### Personal Health behaviors

**Fig. 8:**
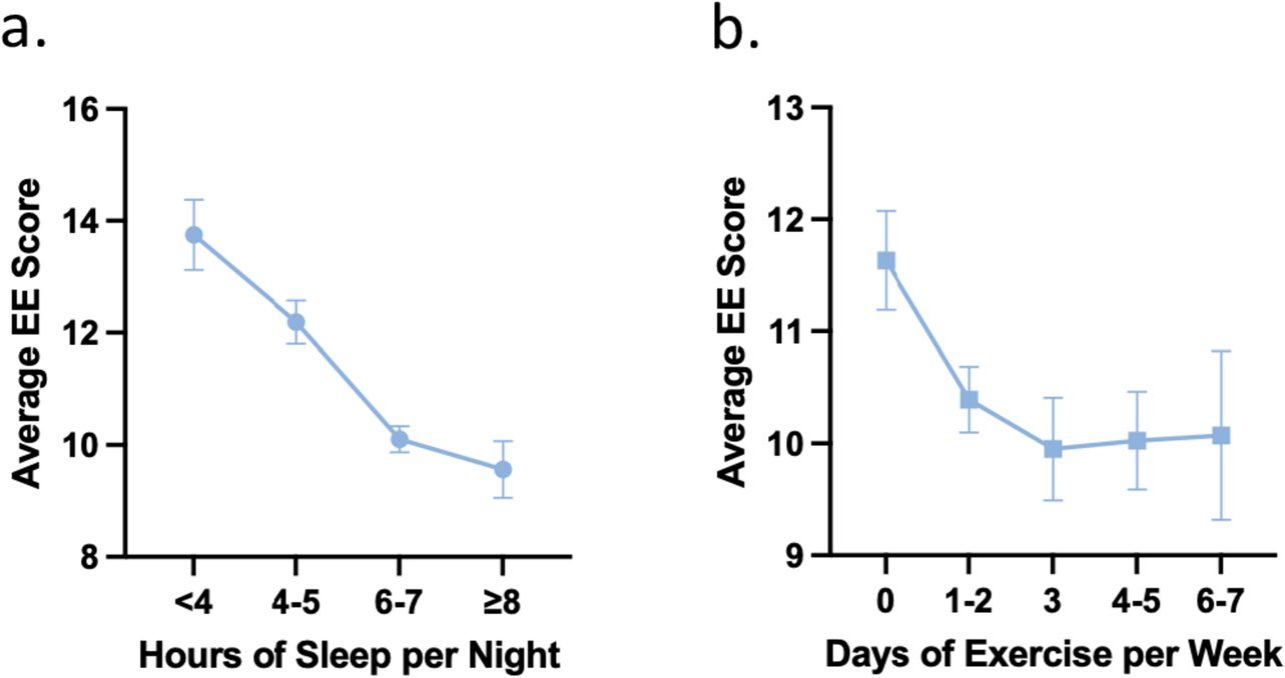
Emotional exhaustion (EE) and personal health habits. **a** Average EE scores and sleep duration per night. Linear trend *p* < 0.0001. **b** Average EE scores and exercise days (of at least 30 consecutive minutes) per week. Linear trend *p* = 0.0071

An analysis of emotional exhaustion and personal health behaviors revealed a decrease in sleep and exercise with increasing emotional exhaustion (Fig. 8).

## Discussion

Our data shows that anxiety was twice as common as depression and that over a third of students screened positive for moderate or severe mood disorder. This finding parallels previous studies that show higher levels of psychological distress among medical students compared to the general population, suggesting that medical training is a time of great personal stress / emotional distress for many students. Notably, our findings showed higher rates of both depression and anxiety than previous studies which may reflect the implementation of our study during the height of the COVID-19 pandemic [12].

Further evaluation by gender revealed higher levels of anxiety, depression, and burnout in female students, a finding supported by previous studies [13, 14]. Among female respondents, almost half had high PHQ-4 scores (> 6), a finding that correlates strongly with functional impairment, disability days, and healthcare use [27]. In comparison, only a quarter of male respondents had similarly high scores. This discrepancy is reflected in the general population where women are twice as likely as men to have anxiety and depression [23].

Notably, there was no variation in anxiety or depression between students in different class years; this did not change even when grouped by preclinical (1st /2nd ) and clinical (3rd /4th ) years, suggesting the transition from an academic to clinical environment has a negligible impact on mood. However, there was a noticeable increase in the level of EE, a strong predictor of burnout, as students transitioned from first to second year [26]. This may be due to the increased pressure to study in preparation for the first board exam in addition to previous obligations from first year. Interestingly, the increased rates of EE did not continue into third year despite the need for preparation for the second board exam. This might suggest that as students advance in training, they acquire the experience and resilience necessary to better manage the increased stress that comes with increased expectations. From third to fourth year, the incidence of EE did not change significantly. However, it should be noted that the survey for fourth year students was distributed at the end of the academic year, after the residency match, a notoriously stressful process, had concluded.

We found that the PBL curriculum, which is based on working in small groups, may be less stressful for medical students than the traditional LBL curriculum. Although outside the cutoff for statistical significance, anxiety and depression tended to be lower in PBL students than LBL students. Anxiety and depression are strongly associated with burnout which when represented as EE, was significantly lower among PBL students [28]. While novel, this finding may not be entirely surprising since previous studies have suggested that PBL provides an improved educational environment compared to LBL [22]. Compared to LBL, which is characterized by large class sizes and one way dissemination of information by the instructor, PBL offers small group sizes and collaboration led by a facilitator. These factors may positively influence students’ perceptions of their learning environment and provide regular interpersonal engagement which may translate to an overall better mindset as they progress through medical school. However, these observations must be viewed in light of an inherent selection bias as students generally self-select into the two curriculum tracks. However, PBL spots are limited, therefore not all accepted students may get their preference. It is possible that students who are uncomfortable working in more closely interacting small groups may have selected LBL; conversely, students with greater confidence in their abilities may have chosen the PBL curriculum. These personal characteristics could very well contribute to the curriculum differences observed in this study. Therefore, this confounding factor makes it difficult to assign an improved educational environment in PBL as the sole factor for the differences observed.

When evaluating anxiety and depression by both curriculum and gender, female students had similar rates of anxiety and depression regardless of whether they were in the PBL or LBL curriculum. The finding suggests that the influence of gender on mood outcomes is stronger than any curriculum difference or potential selection bias.

Another interesting finding in this study was the inverse relationship between EE and sleep and exercise. This raises the question of whether EE, as a component of burnout, leads to decreased sleep and exercise or vice-versa. One study identified too little sleep as a predominant factor in the development of burnout, supporting the idea that adequate sleep is important in preventing burnout [29]. However, there is likely a bidirectional relationship between sleep and burnout. For medical students, establishing good sleep habits early on can help reduce burnout, potentially reducing the risk of making detrimental medical mistakes in the future [30].

This study supports prior studies documenting the prevalence of anxiety, depression, and burnout among medical students [13] which, as Dyrbye et al. pointed out previously, can contribute to suicidal ideation [9]. The potential consequences warrant investigation into reducing such prevalence. We highlight new evidence suggesting that students completing their preclinical years of medical school under the PBL curriculum have less EE than students in the traditional LBL curriculum. This previously undescribed result may represent an additional benefit of the PBL curriculum model which applicants might consider when making their medical school choices. Schools with only an LBL curriculum may seek to offer more PBL-like structure or an entire PBL curricular track. However, the true benefit of PBL cannot be fully assessed without controlling for self-selection.

### Limitations

It is recognized that the study has an inherent selection bias as a major limitation since the LBL and PBL samples are nonrandom. Another limitation of this study is the subjective nature of some of the survey questions and corresponding answers. A further limitation is the smaller sample number of PBL respondents compared to LBL respondents. This is a consequence of the already smaller PBL class sizes. Despite these limitations, the study is the first of its kind where a comparison between two curricula is conducted within a single institution and lays the foundation for future analyses.

## Data Availability

The dataset supporting the conclusions of this article is available in the Figshare repository, 10.6084/m9.figshare.22247161.v1.
